# GLP‐1 receptor agonists, metabolic syndrome, and Alzheimer's disease: Lessons and opportunities from the EVOKE trials

**DOI:** 10.1111/jne.70246

**Published:** 2026-08-11

**Authors:** Pragati Gupta, Valeria Pozzilli, Anastasia De Giovanni, Elisabetta Sapio, Francesco Motolese, Fioravante Capone, Paolo Pozzilli

**Affiliations:** ^1^ Barts and The London School of Medicine and Dentistry Queen Mary University of London London UK; ^2^ Department of Medicine and Surgery, Unit of Neurology, Neurophysiology, Neurobiology and Psychiatry Università Campus Bio‐Medico di Roma Rome Italy; ^3^ Queen Square Multiple Sclerosis Centre, Department of Neuroinflammation, UCL Queen Square Institute of Neurology, Faculty of Brain Sciences University College London London UK; ^4^ Department of Engineering Campus Bio‐Medico University Rome Italy; ^5^ Department of Neurology Fondazione Policlinico Universitario Campus Bio‐Medico Rome Italy; ^6^ Centre of Immunobiology The Blizard Institute, Queen Mary University of London London UK; ^7^ Unit of Thyroid and Metabolic Diseases University Campus Bio‐Medico Rome Italy

**Keywords:** Alzheimer's disease, cognitive decline, GLP‐1 receptor agonists, insulin resistance, metabolic syndrome

## Abstract

Metabolic syndrome and Alzheimer's disease (AD) are increasingly recognised as interconnected, sharing vascular, metabolic and inflammatory pathways that accelerate brain ageing and cognitive decline. This review critically examines the evidence that glucagon‐like peptide‐1 receptor agonists (GLP‐1RAs), initially developed for diabetes and obesity, may influence AD related pathways or reduce AD risk in metabolically vulnerable populations. Metabolic syndrome and AD related cognitive impairment share biological mechanisms including vascular damage, insulin resistance and chronic inflammation, suggesting that therapies targeting metabolic dysfunction could influence neurological outcomes. GLP‐1RAs are notable for combining systemic benefits, like weight loss, improved glycaemic control, and reduced cardiovascular risk, with possible but incompletely established central nervous system effects. Experimental studies suggest that some GLP‐1RAs may reduce amyloid and tau pathology, support mitochondrial function, and limit neuroinflammation, although recent preclinical studies and limited brain penetrance argue against a direct neuroprotective effect. Observational data suggest lower dementia rates among users compared with other antidiabetic treatments. However, the recent EVOKE and EVOKE + trials showed that oral semaglutide did not slow clinical progression in early symptomatic AD, despite positive biomarker effects. This highlights the challenge of translating biological plausibility into meaningful clinical benefit and refocuses attention on disease stage, target population, and mechanism. Beyond summarising evidence, this review advocates a more cautious and mechanism specific framework: preserving cognitive health may require addressing systemic metabolic dysfunction rather than targeting the brain alone. GLP‐1 RAs have failed to show an effect in symptomatic AD, but their effects on metabolism might still be beneficial at earlier stages, such as the preclinical phase of AD. Future studies should determine whether earlier intervention in metabolically enriched groups can alter AD related cognitive, vascular, and biomarker trajectories.

## 1 INTRODUCTION

The global epidemics of metabolic syndrome (MetS) and Alzheimer's disease (AD) represent two of the most pressing challenges to public health in the 21st century and understanding how metabolic dysfunction may influence AD related neurodegeneration is now a vital focus for preventing disease. MetS affects over a billion people worldwide and constitutes a cluster of conditions including central obesity, insulin resistance, arterial hypertension, and dyslipidaemia. It establishes a systemic state that is thought to accelerate brain ageing and to favour neurodegeneration.^1^ Concurrently, a growing body of epidemiological work links MetS and its components to an increased risk of cognitive decline and dementia.^2^ Recent meta‐analyses report a modest but consistent association of incident dementia among those with MetS, with the risk being particularly pronounced when metabolic disturbances are present in midlife.^2^ Although many epidemiological studies report all cause dementia rather than biomarker defined AD, these data provide an important rationale for examining metabolic dysfunction as a potentially modifiable contributor to AD risk and progression.

This established epidemiological link is underpinned by biological mechanisms. As detailed in this review, MetS drives brain pathology through a triad of intertwined pathways: vascular injury, impaired brain insulin signalling, and systemic inflammation that fuels neuroinflammation and neurodegeneration.^3^, ^4^, ^5^ Together, these processes may promote AD pathology, vascular impairment, and mixed Alzheimer's/vascular pathology, which is common in late‐life cognitive impairment. Despite this compelling pathophysiology, strategies to mitigate AD related cognitive decline by targeting metabolic health remain underexplored in clinical practice.

It is within this context that glucagon‐like peptide‐1 receptor agonists (GLP‐1RAs) emerge as a uniquely promising therapeutic opportunity. Originally developed for type 2 diabetes, these agents have revolutionised metabolic care by producing profound weight loss, improving glycaemic control, and reducing major cardiovascular events.^6^ Their multifaceted mechanisms of action, encompassing systemic vascular protection, reduction of inflammation, and possible central nervous system (CNS) mediated effects, intersect with the pathways linking MetS to AD. This positions GLP‐1RAs as a potential ‘bridge therap’ to target shared metabolic and neurodegenerative pathways.

Although this review focuses on GLP‐1RAs, other metabolic therapies, including metformin and sodium–glucose cotransporter‐2 inhibitors, are also relevant to dementia and AD research.^7^, ^8^ GLP‐1RAs are emphasised here because they combine broad metabolic and vascular effects with possible CNS mediated mechanisms and, uniquely, have now been tested in dedicated symptomatic AD trials, including ELAD, EVOKE, and EVOKE+.^9^, ^10^

The aim of this narrative review is to synthesise the current evidence connecting MetS to AD and to examine the hypothesis that GLP‐1RAs could modify AD related pathways or reduce future AD risk in metabolically vulnerable populations. We will explore the vascular, metabolic, and inflammatory mechanisms underpinning this relationship, critically appraise the growing epidemiological signals from real‐world studies, and examine the results of recent pivotal randomised controlled trials, including the recently published EVOKE and EVOKE+ trials, to assess whether this promising class of drugs can alter the trajectory of AD related cognitive decline.

## 2 CONNECTIONS BETWEEN MetS AND AD

### 2.1 Vascular mechanisms and mixed pathology

MetS affects the brain through intertwined vascular, metabolic, and inflammatory mechanisms that together may accelerate AD‐related cognitive decline and contribute to mixed AD/vascular pathology.^5^ Chronic exposure to hypertension, dyslipidaemia, central adiposity, and insulin resistance promotes large‐ and small‐vessel pathology, endothelial dysfunction, and arterial stiffness. The resulting burden of white matter damage, including hyperintensities, lacunes, and microinfarcts, causes vascular cognitive impairment and often coexists with AD‐type pathology in one‐fifth to one‐third of dementia cases. Thus, MetS amplifies both the risk of pure vascular dementia and the vascular contribution to AD.^5^

Although estimates of the prevalence of mixed AD and vascular pathology vary widely across studies, ranging from approximately 30%–80% and increasing with age, autopsy series consistently demonstrate that most older adults with dementia harbour both AD neuropathological change and cerebrovascular disease, while pure AD is seen in 30%–45% of cases.^11^, ^12^ Moreover, both vascular pathology and AD neuropathologic change are independently more frequent with advancing age.^13^ While vascular and AD pathology are both common, the link between MetS and AD is not solely vascular. Even subtle vascular injury, for example, minor blood–brain barrier (BBB) leakage or modest reductions in cerebral blood flow, can impair perivascular/glymphatic and BBB‐mediated clearance of amyloid‐*β* and tau, thereby accelerating the accumulation of hallmark AD pathologies.^14^

### 2.2 Metabolic and insulin‐signalling dysfunction

Beyond these vascular changes, impairment of metabolic signalling within the brain could contribute to AD related neurodegeneration. Insulin resistance extends beyond peripheral tissues: neurons exposed to chronic hyperinsulinemia exhibit reduced glucose uptake, altered mitochondrial function, and greater vulnerability to oxidative stress.^3^ Recent AD mouse model studies show that impaired insulin signalling in astrocytes and microglia can disturb amyloid beta uptake, glial metabolism, neuroinflammatory response, and AD‐like pathology.^15^, ^16^ Neuroimaging and cerebrospinal‐fluid studies demonstrate disrupted insulin‐signalling cascades and reduced cerebral glucose metabolism in individuals with type 2 diabetes and AD, supporting brain insulin resistance as a mechanistic bridge between systemic metabolic dysfunction and hallmark AD pathology.^3^ Yet few human studies simultaneously track peripheral metabolic change, brain insulin biomarkers, glial or inflammatory markers, AD biomarkers, and longitudinal cognition, which limits causal inference.

### 2.3 Inflammation and oxidative stress in AD

Inflammation and oxidative stress further reinforce this metabolic‐vascular interplay. MetS creates a chronic pro‐inflammatory environment, partly driven by visceral adiposity, altered adipokine signalling, adipose tissue macrophage infiltration and increased circulating inflammatory mediators such as IL‐6, TNF‐*α* and CRP.^4^, ^17^ These peripheral inflammatory signals may affect the brain through endothelial activation, BBB dysfunction, and immune to brain signalling. In AD, this is particularly relevant because neuroinflammation is closely linked to amyloid‐β deposition, tau pathology, and neuronal injury.^17^, ^18^, ^19^

Microglia and astrocytes are central mediators of this process. Microglial activation may initially support amyloid clearance, but sustained or dysregulated activation can promote cytokine release, oxidative stress and complement activation.^18^, ^19^ Astrocytic dysfunction can impair metabolic support, BBB integrity, and glymphatic/perivascular clearance. These processes feedback to worsen vascular and insulin signalling abnormalities, producing a self‐propagating cycle of metabolic, inflammatory, and neural damage.^4^, ^17^

These inflammatory pathways are relevant to GLP‐1RAs because preclinical AD models suggest that GLP‐1 receptor agonism may modulate neuroinflammation and oxidative stress, although whether these effects are mediated by direct CNS receptor activation, systemic anti‐inflammatory effects, vascular improvement, or a combination of mechanisms remains uncertain.^20^

### 2.4 Timing of obesity and the obesity paradox

Complicating this model, the relationship between adiposity and AD or dementia risk is time‐dependent. Midlife obesity is consistently associated with increased late‐life dementia risk, whereas higher BMI measured in late life is sometimes neutral or even inversely associated with dementia risk; this is called the ‘obesity paradox’.^21^ This pattern likely reflects reverse causation and under‐reporting of age of obesity onset, a key but rarely reported variable.^21^ Late‐onset obesity may have different implications for cerebrovascular versus neurodegenerative mechanisms, underscoring the need for longitudinal cohorts that record obesity onset and duration. A similar timing issue may apply to type 2 diabetes: midlife diabetes is generally associated with increased later life cognitive decline or dementia risk, whereas late‐life associations may be attenuated by survival bias, competing mortality, disease duration, treatment exposure, and reverse causation.^22^, ^23^

### 2.5 Genetic evidence linking metabolic pathways and AD

Genetic evidence provides additional support for overlap between metabolic and AD related pathways. Large AD GWAS have identified risk loci involving lipid metabolism, immune function and microglial biology. Cross‐trait GWAS analyses have reported shared genetic architecture between AD and metabolic traits, including fasting glucose, fasting insulin, and HDL cholesterol.^24^, ^25^ However, Mendelian randomisation studies of type 2 diabetes and AD have produced mixed or non‐causal findings, indicating that epidemiological associations may not reflect a single causal pathway, but may depend on timing, vascular burden, genetic susceptibility and the specific metabolic pathway involved.^26^

Collectively, evidence supports a multifactorial model in which MetS may contribute to AD related cognitive decline and mixed AD/vascular pathology through vascular injury, brain insulin‐signalling dysfunction, and systemic‐to‐central inflammation (Table 1). Understanding how these pathways interact is critical for designing interventions that target metabolic health and evaluate AD related cognitive and biomarker outcomes, which is an approach explored in emerging trials of GLP‐1 receptor agonists (GLP1RAs).

**TABLE 1:** Summary of evidence linking GLP‐1 receptor agonists (GLP‐1RAs) to Alzheimer's disease (AD).

Dimension	Key insights	Representative evidence	Implications
Metabolic rationale	Metabolic syndrome may contribute to AD via vascular injury, insulin resistance, and systemic inflammation.	Mechanistic and epidemiological studies^1^, ^2^, ^4^	Targeting metabolic dysfunction may modify dementia risk, but causality remains incompletely established.
Mechanisms of GLP‐1RAs	Improve cardiometabolic health and may influence AD relevant pathways through vascular/metabolic effects and potential CNS mediated mechanisms.	Animal models and translational data^32^, ^33^	GLP‐1RAs provide a model for testing metabolic modulation in AD, but CNS engagement should not be equated with direct parenchymal neuroprotection.
Human observational evidence	GLP‐1RA users show lower all cause dementia incidence versus other antidiabetic agents.	Cohort and meta‐analysis^41^, ^43^	Signals possible real‐world cognitive benefit; but is vulnerable to residual confounding and outcome misclassification.
Randomised clinical trials	ELAD study suggested a possible executive function signal with liraglutide, a small exenatide pilot study was neutral; EVOKE and EVOKE+ showed no clinical benefit of oral semaglutide in early symptomatic AD.	ELAD; EVOKE and EVOKE+^9^, ^10^	Current RCT evidence does not establish GLP‐1Ras as therapies for symptomatic AD.
Pharmacologic nuances	BBB penetration and CNS exposure vary across GLP‐1RA; findings from one agent may not directly apply to another.	Translational pharmacology studies^31^, ^58^	Agent selection matters for AD trials.

*Note*: This table summarises key mechanistic, clinical, and translational evidence linking GLP‐1RAs to AD.

## 3 GLP‐1 RECEPTOR AGONISTS BRIDGING MetS AND DEMENTIA

As addressed in the introduction, GLP‐1 RAs have transformed metabolic medicine. More recently, a growing body of translational and clinical evidence suggests that the benefits of GLP‐1RAs may extend beyond peripheral metabolism to pathways relevant to brain ageing and AD related cognitive decline, with observational studies showing associations between GLP1‐RA use and lower incidence of AD and related dementias in T2D.^27^ Their mechanisms intersect with the metabolic and vascular pathways that may contribute to AD pathology, vascular cognitive impairment, and mixed AD/vascular disease, making them a clinical relevant model for testing whether metabolic modulation can influence AD related outcomes.

### 3.1 Peripheral and potential central mechanisms of action of GLP‐1 RAs

**FIGURE 1:**
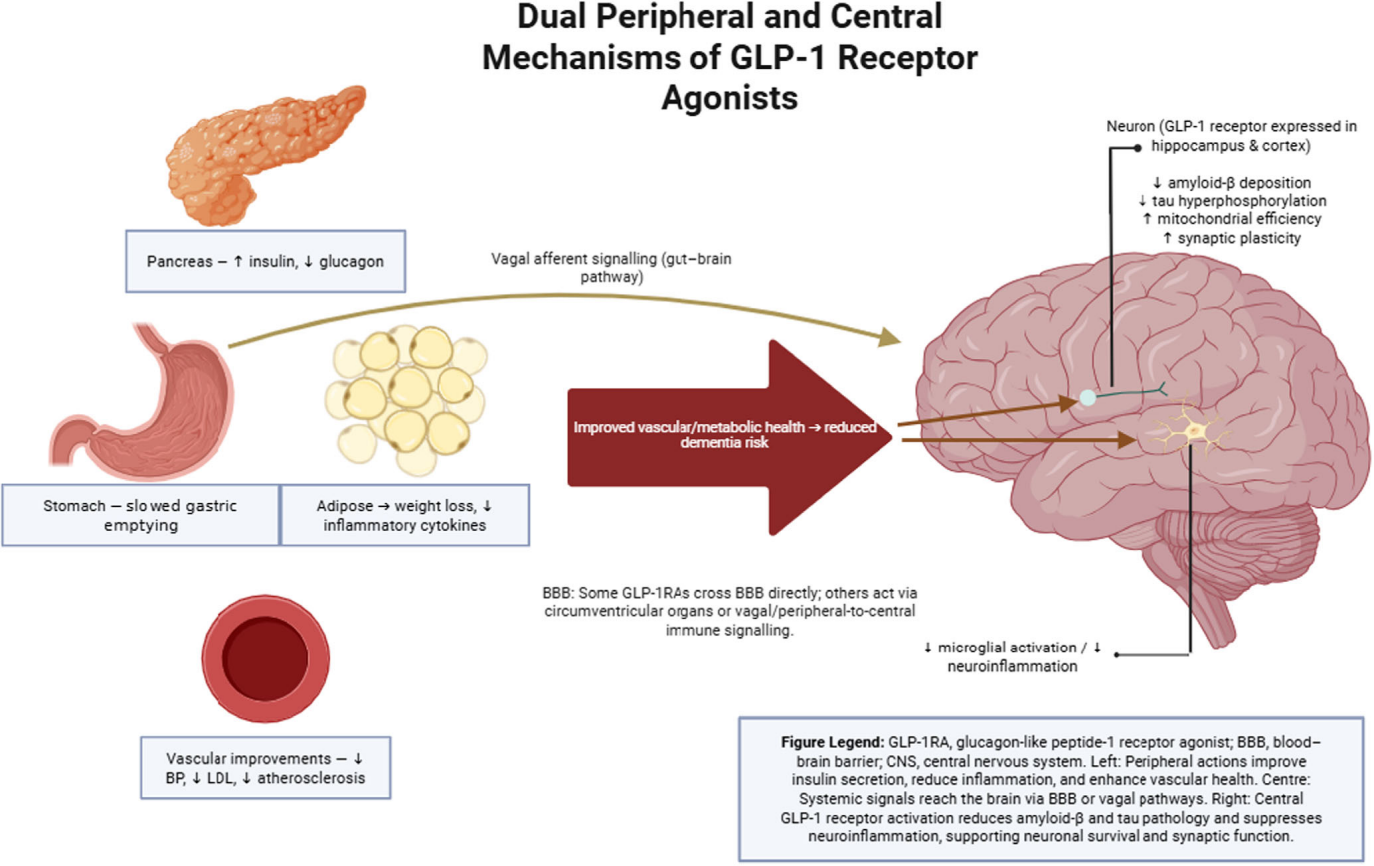
Dual peripheral and central mechanisms of GLP‐1 receptor agonists linking metabolic health to neuroprotection.

GLP‐1RAs act through both systemic and potential CNS mechanisms (Figure 1).^6^ Peripherally, they enhance insulin secretion, suppress glucagon release, and slow gastric emptying, while appetite reduction is mediated largely through central and gut‐brain signalling pathways^6^ GLP‐1 receptors are expressed in several peripheral tissues and cell types, including pancreatic islets, the gastrointestinal tract, cardiovascular and renal tissues, and immune cells. Emerging evidence also suggests GLP‐1 receptor expression in the olfactory system and the eye.^28^ This widespread peripheral and gut‐brain receptor distribution provides a biological basis for systemic metabolic, vascular and inflammatory effects of GLP‐1RAs. These systemic effects are clinically meaningful: sustained reductions in blood pressure, low‐density lipoprotein cholesterol, and low inflammatory markers translate into reduced vascular risk, which is one of the dominant mediators linking MetS to AD related cognitive decline and mixed vascular pathology.^6^ The STEP 1 trial demonstrated that weekly semaglutide 2.4 mg produced an average 14.9% weight loss and broad metabolic benefits in people with obesity.^29^ The recent SELECT trial extended these findings by showing that semaglutide reduced major adverse cardiovascular events by 20% in individuals with obesity but without diabetes.^30^ These outcomes underscore that GLP‐1RAs exert substantial vascular protection, a mechanism that may plausibly influence cerebrovascular pathways relevant to AD expression and progression.

Beyond these peripheral effects, compelling experimental work demonstrates that GLP‐1RAs may influence CNS pathways, although the extent to which clinically used agents access learning and memory related parenchymal regions remains uncertain.^31^ GLP‐1 receptors have been described in brain regions implicated in learning and memory, including the hippocampus and cortex, particularly in preclinical studies, but receptor expression does not necessarily imply effective drug exposure after peripheral administration.^31^, ^32^ This distinction is important because several clinically used GLP‐1RAs have limited brain access; therefore, effects on CNS inflammatory cells could reflect indirect rather than direct drug action. In preclinical AD models, earlier generation GLP‐1RAs reduced amyloid‐*β* deposition, inhibited tau hyperphosphorylation, improved mitochondrial efficiency, and restored synaptic plasticity.^33^ A 2023 meta‐analysis of 26 animal studies reported that GLP‐1RAs improved cognitive performance and reduced AD‐like pathology in rodents.^33^ However, preclinical evidence is not uniformly positive. In a recent study on AD mouse models, semaglutide and tirzepatide produced expected metabolic effects, including reduced glycaemia and weight loss, but did not improve cognition or behavioural outcomes and did not reduce amyloid plaque burden or neuroinflammation.^34^ This disparity in preclinical findings provides an important mechanistic context for the negative EVOKE and EVOKE+ results: GLP‐1RA effects on AD‐like pathology may be agent and timing dependent.

### 3.2 From metabolic trials to the AD hypotheses

The success of GLP‐1RAs in obesity and cardiovascular medicine fuels the hypothesis that the same interventions might alter AD related cognitive trajectories. Cardiovascular disease and cerebrovascular pathology share overlapping mechanisms with cognitive decline, for example, arterial stiffness, endothelial dysfunction, microvascular rarefaction, and chronic inflammation, several of which may be improved by GLP‐1RA therapy.^35^ By improving these systemic determinants, GLP‐1RAs could reduce the vascular contributions to cognitive impairment and dementia (VCID) and indirectly slow AD related pathology or its clinical expression, which is often compounded by vascular injury.^35^

The neurovascular relevance of GLP‐1RAs is supported by cardiovascular and stroke trials. In SELECT, semaglutide reduced major adverse cardiovascular events, including nonfatal stroke, in patients with overweight or obesity and established cardiovascular disease without diabetes.^30^ Dedicated stroke trials have also reported preliminary vascular signals: liraglutide reduced recurrent stroke in the LAMP trial of minor ischaemic stroke or high‐risk TIA with type 2 diabetes,^36^ while semaglutide was safe in the GALLOP phase 2 large‐vessel occlusion trial and showed benefit in patients not receiving intravenous thrombolysis.^37^ Other studies remain registered or ongoing, including ASSET in acute ischaemic stroke^38^ and an exenatide cerebral blood‐flow study.^39^

Equally intriguing is their potential to modify brain insulin resistance. Insulin in the brain regulates synaptic plasticity and neuronal survival; its dysregulation has been described as ‘type 3 diabetes’. GLP‐1RAs improve peripheral insulin sensitivity and may restore insulin signalling cascades within the CNS, attenuating the amyloidogenic and tau‐phosphorylating consequences of insulin resistance.^40^ Human studies have documented impaired cerebral glucose metabolism and altered insulin receptor substrate (IRS‐1) phosphorylation in both T2D and AD, linking the two disorders mechanistically.^40^

### 3.3 Human observational evidence

Real‐world cohorts have observed lower all‐cause dementia incidence among GLP‐1RA users compared with other antidiabetic therapies.^41^ In a large 2025 Taiwanese retrospective cohort study of over 100,000 patients with type 2 diabetes and mean follow‐up period of 41 months, GLP‐1RA use was associated with a significant reduction in dementia risk. After multivariable adjustment for comorbidities and concurrent medications, users exhibited an 8% lower risk of incident dementia compared to non‐users (adjusted Hazard Ratio [aHR] 0.92, 95% CI 0.88–0.97).^41^ Similarly, a large retrospective cohort study of US electronic health records found that the use of semaglutide or tirzepatide was associated with a significantly reduced incidence of dementia (HR 0.63) and ischemic stroke (HR 0.81) compared to other antidiabetic drugs in patients with type 2 diabetes and obesity.^42^ Since these studies reported clinical diagnoses rather than biomarker defined AD, they should be interpreted as evidence for lower clinically coded dementia risk rather than evidence of reduced AD.

Meta‐analyses pooling randomised and observational data echo this signal.^43^ A 2025 systematic review of 23 randomised clinical trials found that GLP‐1RAs were associated with a statistically significant reduced risk of dementia or cognitive impairment.^43^ While encouraging, these findings remain hypothesis generating. Potential confounders include treatment selection bias, disease severity, and healthier‐user effects, emphasising the need for randomised studies with pre‐specified cognitive outcomes and AD biomarkers. Nevertheless, the consistency of directionality across independent populations and different agents provides a rationale for interventional research.

### 3.4 Randomised clinical trials in AD

Before EVOKE and EVOKE+, smaller clinical trials tested GLP‐1RAs in AD. In the phase 2b ELAD trial, daily liraglutide for 52 weeks did not significantly improve cerebral glucose metabolism, the primary endpoint, in non‐diabetic participants with mild‐to‐moderate AD syndrome.^9^ An executive function secondary outcome favoured liraglutide, while measures of activities of daily living and global clinical severity showed no significant differences. Therefore, ELAD suggested a possible domain‐specific cognitive signal but did not establish clinical disease modification. A small phase II pilot of exenatide in early AD reported acceptable safety and tolerability but no clear clinical or cognitive benefit, although its small sample size limits interpretation.^44^

The pivotal phase 3 EVOKE and EVOKE+ trials randomised over 3800 adults with early symptomatic, amyloid positive AD (including mild cognitive impairment or mild dementia), to once daily oral semaglutide (14 mg) or placebo for 104 weeks.^10^

Critically, semaglutide did not meet its primary endpoint, showing no significant difference from placebo in slowing clinical disease progression as measured by the Clinical Dementia Rating‐Sum of Boxes (CDR‐SB) in either trial. Secondary clinical endpoints, including other measures of cognition, daily functioning, and time to progression to dementia, also showed no significant benefit. However, exploratory biomarker analyses revealed a more nuanced picture: the semaglutide group showed nominally significant reductions (around 10%) in key CSF biomarkers of Alzheimer's pathology (pTau181, pTau217) and neuroinflammation (YKL‐40), alongside expected systemic metabolic benefits including weight loss and reduced high‐sensitivity C‐reactive protein (hs‐CRP).^10^

This dissociation between biomarker modulation and lack of clinical benefit in established early AD raises fundamental questions. It may suggest that 2 years of treatment may be insufficient to translate biomarker changes into measurable cognitive preservation in a population already exhibiting amyloid pathology and symptoms. Alternatively, the primary neuroprotective mechanism of GLP‐1RAs may be more relevant to metabolic or vascular pathways earlier in the causal chain, rather than modifying established neurodegenerative trajectories. Accordingly, treating patients in early presymptomatic phases of disease or metabolically at risk populations could produce more evident clinical effects. These results do not negate the strong mechanistic rationale, but temper claims of direct neuroprotection and refocus the outstanding questions on timing, population, and the relative contributions of vascular vs. amyloid pathways, as discussed in the following sections.

## 4 PHARMACOLOGIC NUANCES AND BBB CONSIDERATIONS

### 4.1 BBB penetration, regional CNS engagement and indirect mechanisms

A critical translational question is whether peripherally administered GLP‐1RAs penetrate the brain in sufficient concentrations to produce direct effects in AD relevant parenchymal regions. Early pharmacokinetic work suggested limited BBB permeability for large, acylated molecules like semaglutide and liraglutide, whereas smaller peptides such as exendin‐4 and lixisenatide cross more readily.^45^ More recent work also indicates heterogeneity in regional brain uptake across incretin receptor agonists, reinforcing that CNS exposure is not uniform across agents and brain regions.^31^, ^46^ This distinction is important because evidence of CNS engagement should not be interpreted as evidence that a drug reaches all brain regions involved in learning, memory or AD pathology. In fact, engagement in circumventricular regions including the hypothalamus or periventricular regions does not imply direct exposure of the hippocampus or association cortex.^31^, ^47^

A more plausible model is indirect CNS modulation. Activation of GLP‐1‐responsive circuits in circumventricular or hypothalamic regions could influence hippocampal and cortical pathology through autonomic output, vagal signalling and neuroendocrine regulation.^47^, ^48^ In parallel, peripheral GLP‐1RA effects on body weight, insulin sensitivity, endothelial function, systemic inflammation, and immune‐cell activation could modify cerebrovascular health and BBB integrity. These changes may, in turn, influence amyloid‐*β* and tau clearance, neurovascular coupling, and glymphatic/perivascular drainage in AD relevant regions that are not directly exposed to high drug concentrations.^45^, ^47^

This distinction also affects interpretation of preclinical AD studies. Many positive rodent studies used earlier generation or exendin based GLP‐1RAs, which may have greater brain penetrance than long acting acylated agents such as semaglutide. Preclinical efficacy should therefore be interpreted as agent and context dependent, rather than as proof of a class wide direct parenchymal neuroprotective effect.^31^, ^33^, ^45^

### 4.2 Imaging approaches to study GLP‐1 receptor engagement

Emerging neuroimaging and molecular tools are central to resolving these uncertainties surrounding CNS penetration and receptor engagement. Various strategies have been developed to characterise GLP‐1 receptor distribution, ligand uptake and target engagement, though most remain in early translational stages. While peripheral GLP‐1 imaging methods are relatively advanced, their application to brain tissue is far less established, in part due to challenges such as limited BBB permeability, low receptor density in certain regions, and uncertainty about whether measurable CNS engagement reflects direct access to AD relevant parenchyma.^49^

In vivo approaches provide the most direct means of assessing GLP‐1 receptor activity in living human brain tissue.^50^, ^51^ These techniques allow researchers to map receptor binding, quantify ligand uptake, and observe pharmacodynamic effects in real time.^51^ Among emerging tools, optical imaging techniques have gained attention for their ability to produce high‐resolution spatial maps of GLP‐1 signalling.^52^, ^53^ Despite their promise, these techniques have been used only in preclinical or in vitro settings; therefore, they lack the translational robustness of established imaging techniques such as positron emission tomography (PET).^52^

PET represents one of the most informative imaging techniques for in vivo molecular studies of the human brain.^54^ To analyse GLP‐1 receptor distribution or target engagement, radiotracers that bind specifically to GLP‐1 or its receptor are injected.^50^, ^55^, ^56^ The PET scanner then detects where radioactive tracers accumulate, which may correspond to GLP‐1 binding sites.^51^ This allows PET to visualise active receptor distribution and density in different tissues and potentially, selected brain regions. Nonetheless, this method faces practical and biological limitations, including the poor BBB permeability of some tracer compounds, low receptor density in some CNS regions, and the short half‐life of positron‐emitting isotopes, which requires access to specialised infrastructure such as cyclotrons.

Complementary modalities such as MRI and SPECT may provide additional structural or functional information but lack PET's sensitivity.^57^ Furthermore, multimodal PET imaging (e.g., amyloid‐ or tau‐PET) combined with metabolic assessment (e.g., FDG‐PET) could yield comprehensive insights by linking GLP‐1 receptor engagement with AD pathology, cerebral metabolism and structural change.^57^ Such multimodal approaches may help clarify whether GLP‐1Ras affect AD related molecular, metabolic or vascular pathways, although they remain exploratory rather than tools for AD clinical trials.

### 4.3 Pharmacological heterogeneity and implications for AD trials

The pharmacologic heterogeneity among GLP‐1RAs implies that ‘class effects’ may differ quantitatively and mechanistically.^58^ Factors such as molecular size, lipophilicity, receptor binding affinity, and half‐life can influence not only metabolic potency but also neural exposure. For AD research, agent choice should balance robust systemic efficacy, plausible central engagement, and tolerability in older adults. Semaglutide's extensive cardiovascular and safety data justify its selection for large outcome trials, while shorter‐acting or more brain‐penetrant agents may still be valuable for mechanistic studies requiring measurable brain penetration.^58^

Newer dual agonists that target both GLP‐1 and glucose‐dependent insulinotropic peptide (GIP) receptors, such as tirzepatide, achieve even greater weight loss and glycaemic improvements.^59^ Preclinical work suggests that dual agonism could enhance synaptic plasticity and dampen neuroinflammation, but human AD specific cognitive and biomarker data are pending. Observational work suggests similar protective associations for tirzepatide as for GLP‐1RAs, which could merit the initiation of dedicated cognitive trials.^59^

## 5 THE MEDIATOR PROBLEM: IS WEIGHT LOSS THE DRIVER OF COGNITIVE BENEFIT?

An enduring question in this field is whether any AD related cognitive or biomarker effect from GLP‐1RAs stems primarily from weight loss and cardiometabolic improvement or from direct or indirect CNS mediated mechanisms.^60^ Weight loss itself can improve cerebral perfusion, reduce systemic inflammation, and lower vascular burden; these are all factors that may influence AD clinical outcomes, particularly when vascular pathology coexists. However, preclinical data showing reduced amyloid and tau pathology independent of weight change argue for potential additional mechanisms beyond weight loss alone.^60^

Future studies should incorporate mediation analyses to quantify the contribution of peripheral metabolic versus CNS effects. Such designs could use multimodal biomarkers, for example, amyloid/tau PET, CSF biomarkers, MRI measures of small‐vessel disease and cortical thickness, and circulating inflammatory markers, to dissect causal pathways. Stratified analyses by baseline BMI, obesity‐onset age, APOE genotype, and vascular burden will clarify which subgroups derive the greatest AD cognitive or biomarker benefit.^60^

## 6 UNMET NEEDS AND FUTURE DIRECTIONS

Despite rapid progress in elucidating the metabolic–neurological interface, significant knowledge gaps remain before GLP‐1RAs can be confidently considered as a treatment option for AD related dementia. The recent results from the EVOKE and EVOKE+ trials, which showed no clinical benefit despite biomarker changes in early symptomatic AD, crystalise several of these critical uncertainties and guide future research priorities.^10^ Table 2 summarises the main knowledge gaps and future directions for GLP‐1RA research in dementia, highlighting who, when, and how these therapies might be most effective, as well as opportunities from biomarkers, real‐world data, and digitaldriven monitoring.

**TABLE 2:** Summary of unmet needs and future directions for GLP‐1RA research in Alzheimer's disease (AD).

Theme	Key issues	Recommendations
Target population	Most existing studies focus on adults with obesity or diabetes. The EVOKE trials demonstrated a lack of efficacy in symptomatic, amyloid‐positive AD populations. It remains unclear whether GLP‐1RAs are more relevant earlier in vulnerable populations.	Future research should extend to non‐diabetic individuals with subclinical metabolic dysfunction, such as metabolic syndrome or pre‐diabetes. Primary prevention studies in cognitively normal, midlife adults should be prioritised, and stratification by genetic risk factors, such as APOE ε4 carrier status, may help identify those most likely to benefit.
Timing and disease trajectory	Midlife obesity increases dementia risk, while higher late‐life BMI may appear neutral or protective due to reverse causation. Most studies record BMI at a single time point and rarely consider the age of obesity onset.	Prospective studies should incorporate BMI trajectories and the timing of obesity onset to identify the critical window for GLP‐1RA intervention and to refine causal inference.
Dementia subtypes and mechanisms	Existing datasets often group all‐cause dementia together, obscuring mechanistic differences. Vascular and mixedAD/vascular pathology subgroups are underrepresented, while preclinical data suggest potential effects on amyloid/tau pathways.	Future trials should prespecify AD, vascular and mixed pathology outcomes and include concurrent amyloid/tau and vascular imaging. This approach will help determine whether GLP‐1RAs primarily act through vascular/metabolic pathways, CNS mediated mechanisms or both.
Trial design and endpoints	Most studies have follow‐up periods under 3 years, which is likely insufficient to detect meaningful changes in dementia incidence. Standard cognitive batteries may not capture relevant outcomes.	Large‐scale real‐world data should be leveraged to enable extended follow‐up. Endpoints should include measures sensitive to vascular cognitive impairment and executive dysfunction, such as executive function tests (e.g., Digit Symbol Substitution Test), functional outcomes, and cerebrovascular events. Multimodal biomarkers (plasma, CSF, imaging) should be included to dissect mediating pathways.
Pharmacologic optimisation and CNS engagement	It remains unclear which GLP‐1RAs effectively cross the blood–brain barrier or achieve optimal central receptor occupancy. The limited BBB penetration of oral semaglutide raises questions about central efficacy.	Future studies should evaluate CNS‐penetrant agents, incorporate CSF pharmacokinetics or GLP‐1 receptor PET imaging, and explore dual or triple agonists, such as tirzepatide, which may have enhanced neuroprotective potential.
Safety and implementation	GLP‐1RAs are generally safe, but gastrointestinal side effects, dehydration, and loss of lean mass may be more problematic in older or frail adults. Rapid weight loss may not be universally beneficial, and high drug costs create equity concerns.	Trials and real‐world implementation studies should monitor frailty indices, muscle mass, and side effects in elderly populations. Economic and equity considerations must guide implementation, and pragmatic studies are needed to test integrated metabolic/cognitive care models.
Bridging clinical practice	There is a disconnect between metabolic and cognitive care, with body weight rarely considered when prescribing dementia therapies and cognitive function seldom assessed before prescribing metabolic medications.	Clinicians should integrate brief cognitive assessments, such as the MoCA, when initiating long‐term GLP‐1RA therapy and proactively manage metabolic risk in patients with cognitive concerns. Evidence is needed to determine whether dementia‐risk reduction should become an explicit goal in metabolic guidelines.
Next‐generation trials	Long‐term randomised trials are difficult due to cost, blinding challenges, ethical considerations, and limited mechanistic insight from single trials.	Large‐scale primary prevention trials should enrol midlife adults with cardiometabolic risk factors and follow them for ≥5 years with co‐primary outcomes of cognitive decline and dementia incidence. Adaptive or pragmatic trial designs, EHR‐linked studies, and mediation analyses should be employed to quantify contributions of peripheral metabolic, vascular, inflammatory and CNS mediated mechanisms.

*Note*: This table highlights key gaps and future directions for studying GLP‐1RAs as AD preventative therapies.

### 6.1 Target population: From treatment to prevention

Current evidence is largely drawn from cohorts of adults with obesity or diabetes. The EVOKE and EVOKE+ trials' lack of clinical efficacy in symptomatic, amyloid‐positive AD cohort suggests that perhaps the neuroprotective window for GLP‐1RAs may lie earlier in the disease cascade.^10^ Future research must extend to non‐diabetic individuals with subclinical metabolic dysfunction (MetS, pre‐diabetes) and, crucially, to primary prevention in cognitively unimpaired at‐risk populations (e.g., midlife adults with obesity or insulin resistance). Stratifying by genetic risk factors such as APOE ε4 carrier status may further help identify individuals most likely to benefit from early GLP‐1RA intervention.^61^ Given that insulin resistance clusters well before diabetes onset, this shift is essential for evaluating the true preventative potential of GLP‐1Ras for AD related outcomes rather than their efficacy as symptomatic stage AD therapies.

### 6.2 Timing, trajectory, and the ‘obesity paradox’

Most studies record BMI at a single time point, yet midlife obesity increases dementia risk while late‐life higher BMI may appear neutral or even protective due to reverse causation.^21^ A similar timing problem may apply to type 2 diabetes: midlife diabetes may confer higher cumulative vascular and metabolic exposure, while late life associations with cognitive decline can be weakened or distorted by survival bias, competing mortality, and reverse causation where preclinical cognitive decline may itself alter behaviour and metabolic control.^22^, ^23^ Virtually, no studies have stratified patients by the age of obesity onset. The EVOKE and EVOKE+ results underscore that timing is critical: intervening in established symptomatic AD may be too late. Prospective studies must therefore incorporate BMI trajectories and onset timing to identify the critical window where GLP‐1RA intervention might yield maximal neuroprotection and to refine causal inference beyond reverse causation.

### 6.3 Dementia, vascular burden, and mechanism‐specific effects

Most existing datasets group all‐cause dementia together, obscuring mechanistic nuance. Given the strong vascular benefits of GLP‐1Ras, including reductions in blood pressure, systemic inflammation, and atherosclerotic events, they are likely to exert the greatest relative effect on vascular dementia and on the vascular contributions to mixed AD/vascular pathology, which is a subgroup underrepresented in the EVOKE trials.^10^, ^30^ This is evidenced as <2% had significant small‐vessel pathology.^10^ Conversely, preclinical data points to effects on AD‐specific pathways (amyloid/tau).^62^ The dissociation between reduced biomarkers and lack of cognitive benefit in EVOKE and EVOKE+ raises a key question: is this insufficient in symptomatic stages, or is the primary benefit vascular and thus most relevant for mixed dementia? Resolving this requires future trials that prespecify subtype outcomes and include concurrent amyloid/tau and vascular (e.g., MRI of small‐vessel disease) imaging to stratify treatment response.

### 6.4 Trial design: Duration, endpoints, and biomarkers

Most existing studies have follow‐up periods under 3 years, a timeframe likely insufficient to detect meaningful changes in dementia incidence. While long‐term randomised trials (≥5 years) remain challenging to fund and conduct, the rapidly expanding real world use of GLP‐1RAs offers an opportunity to leverage large‐scale observational data with extended follow up. In this context, endpoint selection must evolve beyond standard Alzheimer's disease cognitive batteries. Real‐world studies should incorporate measures sensitive to vascular cognitive impairment and executive dysfunction, such as incident vascular dementia diagnoses, changes in executive function (e.g., digit symbol substitution test), functional outcomes (activities of daily living), and cerebrovascular events. Additionally, multimodal biomarker approach (plasma, CSF, imaging) is non‐negotiable to dissect mediating pathways, as EVOKE's exploratory analyses exemplify.

### 6.5 Pharmacologic optimisation and CNS engagement

We still lack definitive evidence about which GLP‐1RA crosses the BBB most effectively or yields meaningful target engagement in AD relevant regions. Agent choice should balance systemic efficacy with plausible central engagement, tolerability, and regional CNS exposure. The EVOKE trials' use of oral semaglutide, which has limited BBB penetration, invites us to question whether a CNS‐penetrant agent (e.g., exenatide) may yield different results.^10^ Incorporating CSF pharmacokinetics or GLP‐1 receptor PET imaging into future trial designs is essential to answer this translational question. Furthermore, the role of dual/triple agonists (e.g., tirzepatide) with potentially enhanced neuroprotective profiles merits dedicated trials with AD specific cognitive, vascular, and biomarker outcomes.

### 6.6 Safety, equity, and implementation in ageing populations

While GLP‐1RAs are generally safe, their specific safety profile in older or frail patients requires closer attention. Gastrointestinal side effects, dehydration, and the loss of lean mass warrant vigilant monitoring in elderly populations at risk of sarcopenia or frailty.^63^ Rapid weight loss may not be universally beneficial in late life; trials should incorporate frailty indices and muscle mass endpoints. Furthermore, translating any future AD related cognitive benefit into practice hinges on equity. These medications remain expensive and variably available; guideline updates without affordability solutions risk widening health disparities.^64^ Pragmatic implementation research is needed to test models of integrated metabolic‐cognitive care. Furthermore, formal health‐economic analyses are needed to evaluate whether the substantial cost of long‐term GLP‐1RA therapy could be justified by potential savings from reduced AD related disability, reduced dementia incidence and delayed long‐term care.

### 6.7 Bridging metabolic and cognitive care

At the clinical interface, a disconnect remains between metabolic and cognitive care. Body weight and metabolic status are rarely considered when initiating pharmacotherapy for AD or related dementias, while cognitive function is seldom assessed before prescribing anti‐obesity or antidiabetic agents. To address the bidirectional metabolic‐cognitive link, a pragmatic first step would be to integrate brief cognitive assessments—for example, with screening tools such as the Mini‐Mental State Examination (MMSE) or the Montrel Cognitive Assessment (MoCA)—a into the evaluation of older adults initiating long‐term GLP‐1RA therapy, and conversely, to proactively manage metabolic risk factors in patients with cognitive impairment. A critical, unresolved question is whether AD‐risk reduction or preservation of cognitive function should become an explicit goal in metabolic syndrome and obesity guidelines. Current recommendations such as the ADA Standards of Care, prioritise cardiovascular and glycaemic endpoints and they do not mention cognitive health.^65^ Before such inclusion, two forms of evidence are required: first, randomised data demonstrating that metabolic therapies reduce clinically meaningful cognitive decline or incident AD related cognitive decline or incident dementia; second, pragmatic implementation data showing that adding cognitive assessment to metabolic management does not create harm through overdiagnosis, anxiety, or inequitable access.

### 6.8 Call for a new generation of trials

To definitively answer whether metabolic modulation can prevent AD related cognitive decline, an ideal future trial would be a large‐scale, long‐duration, with a randomised controlled design. The most scientifically compelling design would be a primary prevention study enrolling cognitively normal, midlife adults (e.g., 50–65 years) with cardiometabolic risk factors (e.g., MetS or obesity), assigning them to a GLP‐1RA or placebo, and following them for ≥5 years with co‐primary outcomes of cognitive decline and dementia incidence, supported by multimodal AD and vascular biomarkers.^66^ To enhance efficiency and personalise the approach, such a trial could use enrichment strategies, enrolling individuals at higher biological risk (such as APOE ε4 carriers) and would be explicitly designed to perform mediation analyses.^61^ These analyses would quantify how much of any cognitive benefit is driven by improved peripheral measures (e.g., HbA1c, inflammation) versus CNS mediated biomarker changes (e.g., hippocampal volume, amyloid/tau PET, or small vessel disease markers).

However, such a definitive trial faces profound practical and ethical challenges that must be acknowledged and innovatively addressed. The first is the fundamental problem of blinding; the profound weight loss and gastrointestinal side effects of GLP‐1RAs make true blinding in a long‐term trial virtually impossible. To mitigate this bias, future studies may need to rely on central, blinded adjudication committees for primary cognitive outcomes, employ objective digital biomarkers or neuroimaging as primary endpoints, or use an active comparator.

Ethical issues should also be considered. Withholding a medication with proven, major benefits for cardiovascular disease and obesity from a high‐risk control group for 5 years raises serious ethical questions, especially given the morbidity and mortality associated with these conditions. A more ethical and pragmatic path may involve comparing the GLP‐1RA against today's best standard of care, which includes intensive lifestyle intervention and other available therapies. This would test a more realistic question: whether this new class adds cognitive protection on top of existing management. Furthermore, the high cost of these drugs creates equity issues in trial access and complicates the translation of any positive result into public health policy.

Given these substantial hurdles, the field may need to look beyond the single, perfect randomised trial and embrace alternative designs. Large‐scale pragmatic trials embedded within healthcare systems could compare cognitive outcomes in patients prescribed GLP‐1RAs for standard metabolic indications versus those on other therapies, using existing electronic health records for follow‐up. Adaptive platform trials could also efficiently test GLP‐1 therapies alongside other preventative strategies in a shared at‐risk population.

Ultimately, we may not find a single, flawless study, but rather a coherent multi‐pronged evidence strategy. This would synthesise insights from pragmatic data, ethically sound active‐comparator trials, and deep mechanistic studies. The EVOKE results have given a clear, if disappointing, answer for one specific population: those with early symptomatic Alzheimer's disease. In doing so, they have forcefully turned our attention to the harder, earlier, and more complex question that remains wide open: whether, and how, we can practically and ethically test if modulating metabolism with GLP‐1 RAs in midlife can protect the ageing brain.

## 7 CONCLUSION

The intricate vascular, metabolic, and inflammatory pathways linking MetS to AD related cognitive decline establish a biological foundation for repurposing drugs like GLP‐1 receptor agonists as metabolic modulators in AD relevant populations. However, the recent, pivotal EVOKE and EVOKE+ trials demonstrate that translating this mechanistic plausibility into clinical benefit is profoundly challenging. While oral semaglutide modulated relevant biomarkers, it failed to slow clinical progression in patients with early symptomatic Alzheimer's disease.

GLP‐1Ras are not currently treatment options for symptomatic AD. Their potential relevance may depend critically on disease stage, metabolic phenotype, vascular burden and agent specific pharmacology. Dementia risk reduction remains a compelling and biologically plausible objective of metabolic therapy, but the EVOKE results indicate that such a benefit is not guaranteed and may depend critically on intervening earlier in the disease course.

Scientific societies and funding bodies should support large‐scale, long‐duration studies focused on primary prevention in at risk midlife populations, incorporating cognitive and multimodal biomarker outcomes. Coordinated real‐world registries and nuanced policy analyses are also essential to quantify the potential health system impact of metabolic interventions on brain health.

Ultimately, the convergence of metabolic and neurodegenerative medicine signals a necessary shift in how ageing related diseases are conceptualised. GLP‐1RAs represent a prototype for testing whether drugs developed to address systemic metabolic dysfunction may also influence neurodegeneration. The EVOKE trials have provided a crucial, if sobering, test of this hypothesis in one specific clinical context. Their results do not close the book but rather refine the question: whether modulating metabolism can alter the preclinical, vascular or metabolic contributors to AD related cognitive decline. This evolving framework of metabolic‐cognitive integration, rigorously tested and iteratively refined by both positive and negative trials, will continue to redefine prevention strategies for decades to come.

## AUTHOR CONTRIBUTIONS

**Pragati Gupta:** Writing – original draft; methodology; writing – review and editing. **Paolo Pozzilli:** Conceptualization; investigation; writing – review and editing; data curation; supervision. **Anastasia De Giovanni:** Visualization; writing – review and editing. **Elisabetta Sapio:** Visualization; writing – review and editing. **Francesco Motolese:** Writing – review and editing; validation. **Fioravante Capone:** Methodology; writing – review and editing. **Valeria Pozzilli:** Conceptualization; writing – original draft; methodology; writing – review and editing.

## CONFLICT OF INTEREST STATEMENT

The authors have no conflict of interest to declare pertinent to this review article.

## Data Availability

Data sharing not applicable to this article as no datasets were generated or analysed during the current study.

## References

[1] Izzo C , Di Pietro P , Gambardella J , Carrizzo A . Editorial: the role of metabolic syndrome and disorders in cardiovascular disease, volume II. Front Endocrinol. 2025;16:1639896. doi:10.3389/fendo.2025.1639896 PMC12213395 40607216

[2] Qureshi D , Collister J , Allen NE , Kuźma E , Littlejohns T . Association between metabolic syndrome and risk of incident dementia in UK biobank. Alzheimers Dement. 2024;20(1):447‐458. doi:10.1002/alz.13439 37675869 PMC10916994

[3] Andrade LJDO , Oliveira LMD , Bittencourt AMV , Lourenço LGDC , Oliveira GCMD . Brain insulin resistance and Alzheimer's disease: a systematic review. Dement Neuropsychol. 2024;18:e20230032. doi:10.1590/1980-5764-dn-2023-0032 38425702 PMC10901561

[4] Elzinga SE , Guo K , Turfah A , et al. Metabolic stress and age drive inflammation and cognitive decline in mice and humans. Alzheimer's Dement. 2025;21(3):e70060. doi:10.1002/alz.70060 40110679 PMC11923576

[5] Peng T , Yang Y , Ma J , et al. Dementia and metabolic syndrome: a bibliometric analysis. Front Aging Neurosci. 2024;16:1400589. doi:10.3389/fnagi.2024.1400589 38934020 PMC11199533

[6] Zheng Z , Zong Y , Ma Y , et al. Glucagon‐like peptide‐1 receptor: mechanisms and advances in therapy. Sig Transduct Target Ther. 2024;9(1):234. doi:10.1038/s41392-024-01931-z PMC11408715 39289339

[7] Campbell JM , Stephenson MD , de Courten B , Chapman I , Bellman SM , Aromataris E . Metformin use associated with reduced risk of dementia in patients with diabetes: a systematic review and meta‐analysis. J Alzheimer's Dis. 2018;65(4):1225‐1236. doi:10.3233/JAD-180263 30149446 PMC6218120

[8] Gunawan PY , Gunawan PA , Hariyanto TI . Risk of dementia in patients with diabetes using sodium‐glucose transporter 2 inhibitors (SGLT2i): a systematic review, meta‐analysis, and meta‐regression. Diabetes Ther. 2024;15(3):663‐675. doi:10.1007/s13300-024-01538-1 38340279 PMC10942948

[9] Edison P , Femminella GD , Ritchie C , et al. Liraglutide in mild to moderate Alzheimer's disease: a phase 2b clinical trial. Nat Med. 2026;32(1):353‐361. doi:10.1038/s41591-025-04106-7 41326666 PMC12823385

[10] Cummings JL , Atri A , Sano M , et al. Efficacy and safety of oral semaglutide 14 mg (flexible dose) in early‐stage symptomatic Alzheimer's disease (evoke and evoke+): two phase 3, randomised, placebo‐controlled trials. Lancet. 2026;407(10544):2167‐2179. doi:10.1016/S0140-6736(26)00459-9 41865758

[11] Strandberg TE , Tienari PJ , Kivimäki M . Vascular and Alzheimer disease in dementia. Ann Neurol. 2020;87(5):788. doi:10.1002/ana.25715 32154602 PMC7317452

[12] Azarpazhooh MR , Avan A , Cipriano LE , Munoz DG , Sposato LA , Hachinski V . Concomitant vascular and neurodegenerative pathologies double the risk of dementia. Alzheimers Dement. 2018;14(2):148‐156. doi:10.1016/j.jalz.2017.07.755 28974416

[13] Nichols E , Merrick R , Hay SI , et al. The prevalence, correlation, and co‐occurrence of neuropathology in old age: harmonisation of 12 measures across six community‐based autopsy studies of dementia. Lancet Healthy Longev. 2023;4(3):e115‐e125. doi:10.1016/S2666-7568(23)00019-3 36870337 PMC9977689

[14] Preis L , Villringer K , Brosseron F , et al. Assessing blood‐brain barrier dysfunction and its association with Alzheimer's pathology, cognitive impairment and neuroinflammation. Alz Res Therapy. 2024;16(1):172. doi:10.1186/s13195-024-01529-1 PMC11290219 39085945

[15] Chen W , Huang Q , Lazdon EK , et al. Loss of insulin signaling in astrocytes exacerbates Alzheimer‐like phenotypes in a 5xFAD mouse model. Proc Natl Acad Sci USA. 2023;120(21):e2220684120. doi:10.1073/pnas.2220684120 37186836 PMC10214134

[16] Chen W , Liu X , Muñoz VR , Kahn CR . Loss of insulin signaling in microglia impairs cellular uptake of a*β* and neuroinflammatory response exacerbating AD‐like neuropathology. Proc Natl Acad Sci USA. 2025;122(21):e2501527122. doi:10.1073/pnas.2501527122 40388612 PMC12130885

[17] Kinney JW , Bemiller SM , Murtishaw AS , Leisgang AM , Salazar AM , Lamb BT . Inflammation as a central mechanism in Alzheimer's disease. Alzheimers Dement. 2018;4:575‐590. doi:10.1016/j.trci.2018.06.014 PMC6214864 30406177

[18] Heneka MT , Carson MJ , El Khoury J , et al. Neuroinflammation in Alzheimer's disease. Lancet Neurol. 2015;14(4):388‐405. doi:10.1016/S1474-4422(15)70016-5 25792098 PMC5909703

[19] De Strooper B , Karran E . The cellular phase of Alzheimer's disease. Cell. 2016;164(4):603‐615. doi:10.1016/j.cell.2015.12.056 26871627

[20] Du H , Meng X , Yao Y , Xu J . The mechanism and efficacy of GLP‐1 receptor agonists in the treatment of Alzheimer's disease. Front Endocrinol. 2022;13:1033479. doi:10.3389/fendo.2022.1033479 PMC9714676 36465634

[21] Li J , Joshi P , Ang TFA , et al. Mid‐ to late‐life body mass index and dementia risk: 38 years of follow‐up of the Framingham study. Am J Epidemiol. 2021;190(12):2503‐2510. doi:10.1093/aje/kwab096 PMC8796797 33831181

[22] Hu J , Pike JR , Lutsey PL , et al. Age of diabetes diagnosis and lifetime risk of dementia: the atherosclerosis risk in communities (ARIC) study. Diabetes Care. 2024;47(9):1576‐1583. doi:10.2337/dc24-0203 38935599 PMC11362119

[23] Xu W , Qiu C , Gatz M , Pedersen NL , Johansson B , Fratiglioni L . Mid‐ and late‐life diabetes in relation to the risk of dementia. Diabetes. 2009;58(1):71‐77. doi:10.2337/db08-0586 18952836 PMC2606895

[24] Kunkle BW , Grenier‐Boley B , Sims R , et al. Genetic meta‐analysis of diagnosed Alzheimer's disease identifies new risk loci and implicates a*β*, tau, immunity and lipid processing. Nat Genet. 2019;51(3):414‐430. doi:10.1038/s41588-019-0358-2 30820047 PMC6463297

[25] Bellenguez C , Küçükali F , Jansen IE , et al. New insights into the genetic etiology of Alzheimer's disease and related dementias. Nat Genet. 2022;54(4):412‐436. doi:10.1038/s41588-022-01024-z 35379992 PMC9005347

[26] Thomassen JQ , Tolstrup JS , Benn M , Frikke‐Schmidt R . Type‐2 diabetes and risk of dementia: observational and Mendelian randomisation studies in 1 million individuals. Epidemiol Psychiatr Sci. 2020;29:e118. doi:10.1017/S2045796020000347 32326995 PMC7214711

[27] Tang H , Donahoo WT , DeKosky ST , et al. GLP‐1RA and SGLT2i medications for type 2 diabetes and Alzheimer disease and related dementias. JAMA Neurol. 2025;82(5):439. doi:10.1001/jamaneurol.2025.0353 40193118 PMC11976648

[28] Saturnino A , Pozzilli P . Neglected GLP‐1 receptors in the eyes and nose: possible correlation with regulation of appetite. Endocrine. 2026;91(1):130. doi:10.1007/s12020-026-04581-z 41925981

[29] Wilding JPH , Batterham RL , Calanna S , et al. Once‐weekly Semaglutide in adults with overweight or obesity. N Engl J Med. 2021;384(11):989‐1002. doi:10.1056/NEJMoa2032183 33567185

[30] Lincoff AM , Brown‐Frandsen K , Colhoun HM , et al. Semaglutide and cardiovascular outcomes in obesity without diabetes. N Engl J Med. 2023;389(24):2221‐2232. doi:10.1056/NEJMoa2307563 37952131

[31] West J , Li M , Wong S , et al. Are glucagon‐like Peptide‐1 (GLP‐1) receptor agonists central nervous system (CNS) penetrant: a narrative review. Neurol Ther. 2025;14(4):1157‐1166. doi:10.1007/s40120-025-00724-y 40172827 PMC12255637

[32] Urkon M , Ferencz E , Szász JA , et al. Antidiabetic GLP‐1 receptor agonists have neuroprotective properties in experimental animal models of Alzheimer's disease. Pharmaceuticals. 2025;18(5):614. doi:10.3390/ph18050614 40430434 PMC12114801

[33] Kong F , Wu T , Dai J , et al. Glucagon‐like peptide 1 (GLP‐1) receptor agonists in experimental Alzheimer's disease models: a systematic review and meta‐analysis of preclinical studies. Front Pharmacol. 2023;14:1205207. doi:10.3389/fphar.2023.1205207 37771725 PMC10525376

[34] Forny Germano L , Koehler JA , Baggio LL , et al. The GLP‐1 medicines semaglutide and tirzepatide do not alter disease‐related pathology, behaviour or cognitive function in 5XFAD and APP/PS1 mice. Mol Metab. 2024;89:102019. doi:10.1016/j.molmet.2024.102019 39216535 PMC11408156

[35] De Giorgi R , Ghenciulescu A , Yotter C , Taquet M , Koychev I . Glucagon‐like peptide‐1 receptor agonists for major neurocognitive disorders. J Neurol Neurosurg Psychiatry. 2025;96(9):870‐883. doi:10.1136/jnnp-2024-335593 40210453 PMC12418562

[36] Zhu H , Yang B , Lu L , et al. Liraglutide in acute minor ischemic stroke or high‐risk transient ischemic attack with type 2 diabetes: the LAMP randomized clinical trial. JAMA Intern Med. 2026;186(1):46‐54. doi:10.1001/jamainternmed.2025.5684 41182740 PMC12584062

[37] Wang H , Ko H , Leung TW , et al. Glucagon‐like peptide‐1 receptor agonist in large vessel occlusion treated by reperfusion therapy—a phase 2 randomized trial. Nat Commun. 2025;16(1):11274. doi:10.1038/s41467-025-66167-z 41392086 PMC12717234

[38] Aarhus University Hospital . The Safety and Efficacy of Acute Subcutaneous Administration of Semaglutide in Non‐diabetic Patients With Acute Ischemic Stroke: A Multicentre, Phase 2, Prospective, Randomized, Open‐label, Blinded Endpoint Trial [Clinical trial registration] [Internet]. clinicaltrials.gov; 2023 Apr [cited 2026 Jul 10]. Clinical trial registration no.: NCT05630586. Available from: https://clinicaltrials.gov/study/NCT05630586

[39] Kruuse C . The Effect of Glucagon‐like Peptide 1 (GLP‐1) Receptor Agonist on Cerebral Blood Flow Velocity in Non‐Stroke Volunteers (EGRABINS1) [Clinical trial registration] [Internet]. clinicaltrials.gov; 2017 Feb [cited 2026 Jul 10]. Clinical trial registration no.: NCT02838589. Available from: https://clinicaltrials.gov/study/NCT02838589

[40] Talbot K . Brain insulin resistance in Alzheimer's disease and its potential treatment with GLP‐1 analogs. Neurodegen Dis Manage. 2014;4(1):31‐40. doi:10.2217/nmt.13.73 PMC4465775 24640977

[41] Cheng H , Yang S , Liao P , Lee C , Jong G . Impact of glucagon‐like Peptide‐1 receptor agonists on the dementia incidence in patients with type 2 diabetes mellitus: a population‐based longitudinal cohort study. Diabetes Metab Res Rev. 2025;41(5):e70058. doi:10.1002/dmrr.70058 40481813 PMC12145076

[42] Lin HT , Tsai YF , Liao PL , Wei JCC . Neurodegeneration and stroke after Semaglutide and Tirzepatide in patients with diabetes and obesity. JAMA Netw Open. 2025;8(7):e2521016. doi:10.1001/jamanetworkopen.2025.21016 40663350

[43] Seminer A , Mulihano A , O'Brien C , et al. Cardioprotective glucose‐lowering agents and dementia risk: a systematic review and meta‐analysis. JAMA Neurol. 2025;82(5):450. doi:10.1001/jamaneurol.2025.0360 40193122 PMC11976645

[44] Mullins RJ , Mustapic M , Chia CW , et al. A pilot study of Exenatide actions in Alzheimer's disease. Curr Alzheimer Res. 2019;16(8):741‐752. doi:10.2174/1567205016666190913155950 31518224 PMC7476877

[45] Salameh TS , Rhea EM , Talbot K , Banks WA . Brain uptake pharmacokinetics of incretin receptor agonists showing promise as Alzheimer's and Parkinson's disease therapeutics. Biochem Pharmacol. 2020;180:114187. doi:10.1016/j.bcp.2020.114187 32755557 PMC7606641

[46] Abdulhameed N , Babin A , Hansen K , et al. Comparing regional brain uptake of incretin receptor agonists after intranasal delivery in CD‐1 mice and the APP/PS1 mouse model of Alzheimer's disease. Alzheimer's Res Ther. 2024;16(1):173. doi:10.1186/s13195-024-01537-1 39085976 PMC11293113

[47] Trapp S , Brierley DI . Brain GLP‐1 and the regulation of food intake: GLP‐1 action in the brain and its implications for GLP‐1 receptor agonists in obesity treatment. Br J Pharmacol. 2022;179(4):557‐570. doi:10.1111/bph.15638 34323288 PMC8820179

[48] Brierley DI , de Lartigue G . Reappraising the role of the vagus nerve in GLP‐1‐mediated regulation of eating. Br J Pharmacol. 2022;179(4):584‐599. doi:10.1111/bph.15603 34185884 PMC8714868

[49] Xie Y , Wang Y , Pei W , Chen Y . Theranostic in GLP‐1R molecular imaging: challenges and emerging opportunities. Front Mol Biosci. 2023;10:1210347. doi:10.3389/fmolb.2023.1210347 37780209 PMC10540701

[50] Deden LN , Booij J , Grandjean J , et al. Brain imaging of the GLP‐1 receptor in obesity using 68Ga‐NODAGA‐Exendin‐4 PET. Brain Sci. 2021;11(12):1647. doi:10.3390/brainsci11121647 34942949 PMC8699257

[51] Velikyan I , Eriksson O . Advances in GLP‐1 receptor targeting radiolabeled agent development and prospective of theranostics. Theranostics. 2020;10(1):437‐461. doi:10.7150/thno.38366 31903131 PMC6929622

[52] Duffet L , Williams ET , Gresch A , et al. Optical tools for visualizing and controlling human GLP‐1 receptor activation with high spatiotemporal resolution. Elife. 2023;12:RP86628. doi:10.7554/eLife.86628 37265064 PMC10262203

[53] Marvin JS , Shimoda Y , Magloire V , et al. A genetically encoded fluorescent sensor for in vivo imaging of GABA. Nat Methods. 2019;16(8):763‐770. doi:10.1038/s41592-019-0471-2 31308547

[54] Nasrallah I , Dubroff J . An overview of PET neuroimaging. Semin Nucl Med. 2013;43(6):449‐461. doi:10.1053/j.semnuclmed.2013.06.003 24094712

[55] Kiesewetter DO , Gao H , Ma Y , et al. 18F‐radiolabeled analogs of exendin‐4 for PET imaging of GLP‐1 in insulinoma. Eur J Nucl Med Mol Imaging. 2012;39(3):463‐473. doi:10.1007/s00259-011-1980-0 22170321 PMC3617488

[56] Luo Y , Pan Q , Yao S , et al. Glucagon‐like Peptide‐1 receptor PET/CT with 68Ga‐NOTA‐Exendin‐4 for detecting localized Insulinoma: a prospective cohort study. J Nucl Med. 2016;57(5):715‐720. doi:10.2967/jnumed.115.167445 26795291 PMC5227553

[57] Moaket OS , Obaid SE , Obaid FE , et al. GLP‐1 and the degenerating brain: exploring mechanistic insights and therapeutic potential. IJMS. 2025;26(21):10743. doi:10.3390/ijms262110743 41226780 PMC12608514

[58] Min JS , Jo SJ , Lee S , et al. A comprehensive review on the pharmacokinetics and drug–drug interactions of approved GLP‐1 receptor agonists and a dual GLP‐1/GIP receptor agonist. DDDT. 2025;19:3509‐3537. doi:10.2147/DDDT.S506957 40330819 PMC12052016

[59] Alshehri GH , Al‐kuraishy HM , Al‐Gareeb AI , et al. A novel therapeutic prospect: a dual‐acting tirzepatide for Alzheimer's disease. Eur J Pharmacol. 2025;1003:177979. doi:10.1016/j.ejphar.2025.177979 40706971

[60] Teixeira LCR , Luizon MR , Gomes KB . Exploring the role of GLP‐1 receptor agonists in Alzheimer's disease: a review of preclinical and clinical evidence. Receptor. 2025;4(1):2. doi:10.3390/receptors4010002

[61] Hayes AF . Introduction to Mediation, Moderation, and Conditional Process Analysis: a Regression Based Approach. 3rd ed. The Guildford Press; 2022.

[62] Cummings JL , Atri A , Feldman HH , et al. Evoke and evoke+: design of two large‐scale, double‐blind, placebo‐controlled, phase 3 studies evaluating efficacy, safety, and tolerability of semaglutide in early‐stage symptomatic Alzheimer's disease. Alz Res Therapy. 2025;17(1):14. doi:10.1186/s13195-024-01666-7 PMC11708093 39780249

[63] Ceasovschih A , Asaftei A , Lupo MG , et al. Glucagon‐like peptide‐1 receptor agonists and muscle mass effects. Pharmacol Res. 2025;220:107927. doi:10.1016/j.phrs.2025.107927 40858197

[64] Hertzer L , Siegel RM . Glucagon‐like peptide‐1‐receptor agonist treatment of obesity: is it cost‐effective and equitable? J Pediatr. 2025;287:114779. doi:10.1016/j.jpeds.2025.114779 40835048

[65] American Diabetes Association Professional Practice Committee . Introduction and methodology: standards of Care in Diabetes‐2024. Diabetes Care. 2024;47(Supplement_1):S1‐S4. doi:10.2337/dc24-SINT 38078587 PMC10725799

[66] Elias‐Sonnenschein LS , Viechtbauer W , Ramakers IHGB , Verhey FRJ , Visser PJ . Predictive value of APOE‐ 4 allele for progression from MCI to AD‐type dementia: a meta‐analysis. J Neurol Neurosurg Psychiatry. 2011;82(10):1149‐1156. doi:10.1136/jnnp.2010.231555 21493755

